# A Chaperone Trap Contributes to the Onset of Cystic Fibrosis

**DOI:** 10.1371/journal.pone.0037682

**Published:** 2012-05-31

**Authors:** Judith A. Coppinger, Darren M. Hutt, Abbas Razvi, Atanas V. Koulov, Sandra Pankow, John R. Yates, William E. Balch

**Affiliations:** 1 Department of Cell Biology, The Scripps Research Institute, La Jolla, California, United States of America; 2 Department of Molecular Biology, The Scripps Research Institute, La Jolla, California, United States of America; 3 Department of Chemical Physiology, The Scripps Research Institute, La Jolla, California, United States of America; 4 The Skaggs Institute for Chemical Biology, The Scripps Research Institute, La Jolla, California, United States of America; 5 The Institute for Childhood and Neglected Disease, The Scripps Research Institute, La Jolla, California, United States of America; Univ. Med. Center Groningen, Univ. of Groningen, The Netherlands

## Abstract

Protein folding is the primary role of proteostasis network (PN) where chaperone interactions with client proteins determine the success or failure of the folding reaction in the cell. We now address how the Phe508 deletion in the NBD1 domain of the cystic fibrosis (CF) transmembrane conductance regulator (CFTR) protein responsible for cystic fibrosis (CF) impacts the binding of CFTR with cellular chaperones. We applied single ion reaction monitoring mass spectrometry (SRM-MS) to quantitatively characterize the stoichiometry of the heat shock proteins (Hsps) in CFTR folding intermediates *in vivo* and mapped the sites of interaction of the NBD1 domain of CFTR with Hsp90 *in vitro*. Unlike folding of WT-CFTR, we now demonstrate the presence of ΔF508-CFTR in a stalled folding intermediate in stoichiometric association with the core Hsps 40, 70 and 90, referred to as a ‘chaperone trap’. Culturing cells at 30 C resulted in correction of ΔF508-CFTR trafficking and function, restoring the sub-stoichiometric association of core Hsps observed for WT-CFTR. These results support the interpretation that ΔF508-CFTR is restricted to a chaperone-bound folding intermediate, a state that may contribute to its loss of trafficking and increased targeting for degradation. We propose that stalled folding intermediates could define a critical proteostasis pathway branch-point(s) responsible for the loss of function in misfolding diseases as observed in CF.

## Introduction

The proteostasis network (PN) is composed of numerous components that direct the highly variable and adaptable folding and degradative capacity of the cell [1]–[3]. This system includes the stress-inducible heat shock proteins (Hsps) such as the Hsp40, Hsc70/Hsp70 (Hsc/p70) and Hsp90 chaperone/co-chaperone ATPase systems [4]–[10], and the ubiquitin/proteasome degradation system [2], [11]–[14]. While much progress is being made in understanding the role of individual components of the PN in facilitating protein folding, our appreciation of the general operating principles by which these components cooperate, and respond to stress and human disease remains largely unknown.

Misfolding diseases such as cystic fibrosis (CF) arise due to the inability of an altered polypeptide chain sequence to properly engage key PN components, resulting in reduced folding efficiency of mutant proteins [1], [12], [15], [16]. The occurrence of CF disease in the human population is largely caused by deletion of Phe508 (F508) in the cystic fibrosis transmembrane conductance regulator (CFTR) protein (ΔF508-CFTR or ΔF508), leading to its retention in the endoplasmic reticulum (ER) and subsequent degradation by ER-associated degradation (ERAD) [3], [17]–[19]. CFTR is a cAMP-activated chloride channel comprised of two transmembrane domains (TMD) each containing 6 membrane-spanning helices, two cytoplasmic-oriented nucleotide-binding domains (NBDs 1 and 2), and a regulatory domain (R). Substantial evidence demonstrates that failure to deliver ΔF508-CFTR to the cell surface reflects its inability to achieve sufficient thermodynamic stability of the cytoplasmic oriented NBD1 domain missing the Phe508 residue [20]–[32]. This defect limits the ability of additional domains, including the TMD, to interact with NBD1 to form a structure recognized by the ER-associated COPII export machinery for delivery to the plasma membrane [3], [17]–[19], [33], [34]. The loss of CFTR at the cell surface results in dehydration of the epithelial lining of the lung and other tissues through a disruption of Na^+^ and Cl^−^ balance, triggering the progressive pathology characteristic of the disease [3], [35].

The biogenesis of CFTR is regulated by chaperones in the ER lumen [36] and by the cytosolic Hsp70, Hsp90 and their regulatory co-chaperones [3], [17]–[19], [33], [37]. Whereas CFTR folding is dependent on the Hsp90 system [33], [38], [39], its targeting for degradation occurs through a link between the Hsp40-Hsc/p70 chaperone system and the proteasome degradation machinery [40]–[43]. Despite a number of studies that have characterized the role(s) of PN components in the biogenesis of both WT- and ΔF508-CFTR, the binding site for these chaperones and the stoichiometry(s) of potential intermediate folding complexes contributing to disease remain unknown. A quantitative understanding of the composition of folding intermediates and how they differ between WT and mutant proteins, such as ΔF508 CFTR, is critical to begin to understand the principles governing protein-folding networks in the cell, insights that will improve our ability to correct misfolding diseases.

Using single ion reaction monitoring mass spectrometry (SRM-MS) [44], [45], we now show that deletion of F508 in CFTR leads to its accumulation in a folding intermediate complex containing stoichiometric to supra-stoichiometric ratios of Hsp70 and Hsp90 core chaperones. We further show a differential interaction of Hsp90 with the WT- and Δ508-NBD1 *in vitro* that may contribute to differences in the interaction of the Hsp90 system with WT and ΔF508-CFTR *in vivo*. Our combined results address a major question in the chaperone field as to how Hsp90 physically interacts with client proteins involved in human disease in the WT and mutant states. They further provide insights into the mechanisms by which an ER-localized ΔF508-CFTR folding intermediate(s) may contribute to CF disease. We suggest that therapeutic interventions that alter either the composition or function of the PN may improve the stability of the misfolded protein allowing it to progress beyond the stalled folding intermediate(s), and potentially have a major impact on the treatment of this human disease.

## Results

### Validation of SRM-MS as a quantitative tool

The biogenesis of both WT- and ΔF508-CFTR are now recognized, at least qualitatively, to be dependent on the functional cycles of Hsp70 and 90 core chaperone systems, despite a clear difference in their respective trafficking abilities [33], [39]–[43]. Therefore, ΔF508-CFTR provides a unique opportunity to quantitatively determine the properties of folding intermediate(s) that impact disease etiology. Such intermediates are likely to have been missed in the analysis of the wild-type (WT) protein given its rapid progress through these early folding intermediates en route to the functional protein.

**Figure 1:**
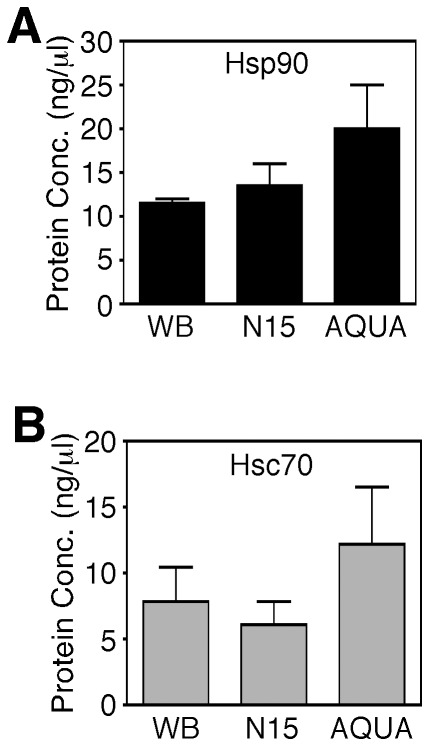
Quantification of CFTR, Hsc70 and Hsp90 in CFTR-containing complexes. **A.** Absolute abundance (ng/µl) of Hsp90 calculated by ^15^N protein labeling, AQUA labeling and Western blotting (WB). **B.** Absolute abundance (ng/µl) of Hsc70 calculated by ^15^N protein labeling, AQUA labeling and Western blotting (WB). In all panels, data is shown as mean ± SD, n≥3.

In order to understand CFTR folding by the PN components differentially associated with WT- and ΔF508-CFTR [33], we used ^15^N-labeled protein standards expressed in *E. coli* and SRM-MS to provide a quantitative analysis of the complexes generated during CFTR biogenesis (**Fig. 1** & **Fig. S1**). Intact ^15^N-labeled proteins allows for the precise characterization of the digestion and processing conditions of the endogenous protein for mass spectrometry. Additionally, SRM-MS allows for analysis of native and internal standard peptides in rapid succession [46]. Using this method, parent masses are selected for MS/MS fragmentation producing unique fragment ions, which are monitored and quantified in the midst of complex mixtures.

Initial validation was performed in order to compare ^15^N-labeled protein based approaches to existing absolute quantification strategies based on AQUA synthetic peptides [47]. Fully labeled chaperone protein standards and labeled synthetic AQUA peptides from Hsc70 or Hsp90 were independently added to 50 µg of HEK293 lysate and traced by SRM-MS under identical chromatographic conditions. The protein abundance of both chaperones determined using ^15^N-labeled proteins was compared to AQUA synthetic peptide standards or quantitative Western blotting (**Fig.** **1**). All three methods correlated well with 13.6±2.9 ng/µl of Hsp90 measured using ^15^N-labeled protein standard (**Fig.** **1A** & **Fig S1A**), 19.5±5 ng/µl using the AQUA synthetic peptide (**Fig.** **1A**) and 11.0±0.8 ng/μl using Western blotting (**Fig.** **1A** **)**. A similar correlation was seen for Hsc70 (**Fig.** **1B** & **Fig S1B**). The abundance of Hsp90 was determined to be ∼1% of total lysate whereas the abundance of Hsc70 was estimated at ∼0.7% of total lysate. The concordance of these values with previous studies suggests a high accuracy in abundance estimation using the ^15^N-labeled SRM-MS approach.

### ΔF508-CFTR is recovered in a chaperone bound intermediate in the ER

**Figure 2:**
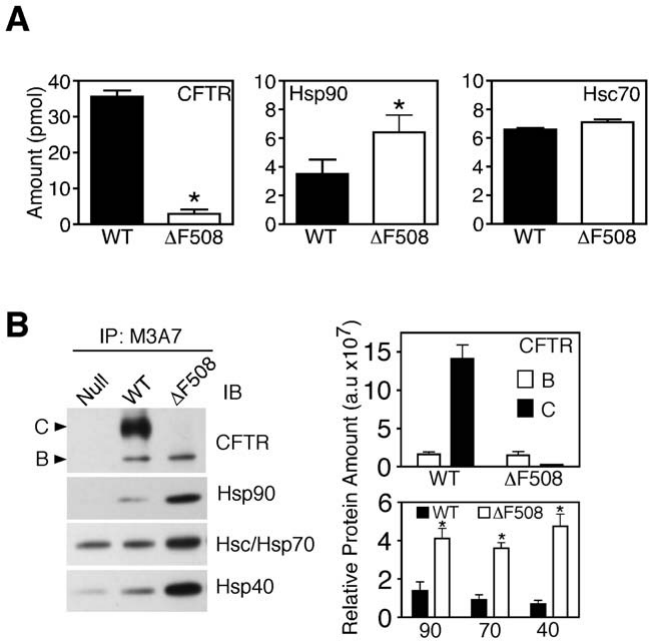
Quantification of WT and ΔF508 CFTR interactions with core chaperones. **A.** The absolute levels of CFTR, Hsp90 and Hsc70, expressed in pmol, in CFTR-containing complexes were determined using the absolute quantification strategy from HEK293 ΔF508-CFTR (white) and WT-CFTR (black) producing cells. **B.** Immunoblot and densitometric analysis for CFTR, Hsp90, Hsc/p70 and Hsp40 from CFTR-containing immunoprecipitates. A representative blot is shown. In the densitometric analysis, the relative protein amount is shown in arbitrary units (a.u.). In all panels, data is shown as mean ± SD, n = 3 and asterisks represent p value <0.05 as determined by two-tailed t-test using the WT sample as the reference.

Transport of CFTR from the ER to the cell surface results in its processing from the band B glycoform, containing two high mannose N-linked oligosaccharides to the band C glycoform, containing Golgi modified glycans that have a slower migration on SDS-PAGE (**Fig.** **2B**). Immunoblot analysis revealed that WT-CFTR accumulates in the band C glycoform at a level that is ∼10-times greater than the ER-associated band B pool (**Fig.** **2B**). However, a similar level of band B is observed in HEK293 cells expressing either WT- or ΔF508-CFTR (**Fig.** **2B**). Given that the rate of nascent synthesis of the two proteins has been shown to be identical [48], these results suggest that the folding machinery supporting the availability of band B is similar for WT and ΔF508. The WT pool proceeds to the COPII export pathway while ΔF508 to degradation. This bifurcation in trafficking decisions is likely a critical event in the disease etiology associated with CF and needs to be understood.

To rigorously quantify the levels of chaperones associated with CFTR at physiological temperature, we first measured the absolute levels, expressed in pmol, of CFTR, Hsc70 and Hsp90 recovered by CFTR immunoprecipitations from HEK293 cell lysates expressing either WT- or ΔF508-CFTR. The complexes were mixed with 5 pmol of AQUA labeled CFTR peptides, (NSILNPINSIR(^13^C^15^N) and GQLLAVAGSTGAGK(^13^C^15^N)), to monitor CFTR concentration and ^15^N-labeled Hsc70 and Hsp90 before digestion. Using SRM-MS we detected a 12-fold increase in the molar amount of total WT-CFTR in the immunoprecipitates as compared to ΔF508-CFTR, reflecting the increased stability of the WT protein (**Fig.** **2A** **&** **Table** **1**), consistent with the protein levels seen by immunoblotting (**Fig.** **2B**). A comparison of the recovered molar levels of Hsc70 and Hsp90 between WT- and ΔF508-CFTR immunoprecipitates reveals a 1.8-fold increase in Hsp90 from ΔF508 immunoprecipitates, compared to WT, and a modest change in Hsc70 (**Fig.** **2A** **&** **Table** **1**). When the absolute molar amounts of recovered chaperones were normalized to the molar recovery of total CFTR in each sample (**Fig.** **2A**), it became apparent that ΔF508-CFTR complexes contain substantially more bound chaperone. A more detailed analysis of the ΔF508 containing band B complexes reveals a stoichiometric to supra-stoichiometric association of chaperones at a molar ratio of 1: 2.4±1.0: 2.2±1.0 (total CFTR: Hsp90: Hsc70) (**Table** **1**). In contrast, chaperone recovery with the total pool of WT-CFTR complexes (band B plus C) is sub-stoichiometric, exhibiting a molar ratio of 1: 0.1±0.01: 0.2±0.01 (total CFTR: Hsp90: Hsc70) (**Table** **1**) illustrating that WT CFTR is able to mature past the Hsp-bound intermediate. Our observations using SRM-MS are in agreement with the increased recovery of Hsp90, Hsc/p70 and Hsp40 seen by immunoblot analysis of co-immunoprecipitations of both WT- and ΔF508-CFTR (**Fig.** **2B**). Furthermore, since these core chaperones do not stably associate with cell-surface localized WT-CFTR [36], the results suggest that the ∼10% of WT-CFTR recovered in the band B glycoform is responsible for the observed chaperone recovery. Therefore, if the molar recovery of chaperones in WT-CFTR complexes is normalized to the band B recovery in the immunoprecipitation, we observe a molar ratio of 1: 1.0±0.3: 1.8±0.4 (band B CFTR: Hsp90: Hsp70), resembling the molar ratio seen for ΔF508-CFTR/chaperone complexes. Given this observation, we conclude that WT-CFTR may also transit through a stoichiometric to supra-stoichiometric Hsp-containing complex prior to ER export. From this perspective, one possibility is that ΔF508-CFTR may be trapped in an on-pathway, stalled folding intermediate rather then an off-pathway, non-productive intermediate. The stalled Hsp–bound folding intermediate may explain the enhanced degradation of ΔF508 observed *in vivo* and its failure to be exported from the ER [17]–[19].

**Table 1:** Stoichiometry of the WT and ΔF508 CFTR interaction with core chaperones.

	ΔF508	Molar Ratio to Total CFTR	WT	Molar Ratio to Total CFTR	Fold Change
**CFTR**	2.9±1.2 pmol	1.0	35.6±1.7 pmol	1.0	+12.3±5.1
**Hsp90**	6.4±1.2 pmol	2.4±1.0	3.5±1.0 pmol	0.1±0.01	−1.8±0.3
**Hsp70**	7.1±0.2 pmol	2.2±1.0	6.6±0.1 pmol	0.2±0.01	−1.1±0.03

Table depicting the absolute amounts of CFTR, Hsp90 and Hsc70, expressed in pmol. Also shown are the molar ratios of chaperones to total ΔF508- or WT-CFTR. The fold change in the absolute amounts of CFTR, Hsp90 and Hsc70, expressed in pmol, relative to ΔF508-CFTR is shown in the final column.

### Resolving the Hsp-bound stalled folding intermediate by reduced temperature

The ΔF508-CFTR mutant is a temperature-sensitive variant [49] that achieves productive trafficking and function when cells are transferred to a permissive temperature (30°C), a condition known to stabilize the fold [26] and reduce Hsc/p70-dependent ERAD [36], [40], [43], [50], [51]. Since the mechanism by which reduced temperature achieves stabilization and transport is unknown, we explored the effect of reduced temperature on the stoichiometry of ΔF508-CFTR intermediates characterized above. In order to eliminate the possibility that degradation is the rate-limiting step, we examined the effect of the proteasomal inhibitor MG132 on ΔF508-CFTR export. While MG132 prevented CFTR degradation at 37°C, as reported previously [38], [52], it did not result in an increase in trafficking, determined by the ratio of band C to band B (C/B), a putative measure of export efficiency from the ER (**Fig. S2**). Thus, CFTR trafficking out of the ER is not limited by the availability of the nascent protein.

**Figure 3:**
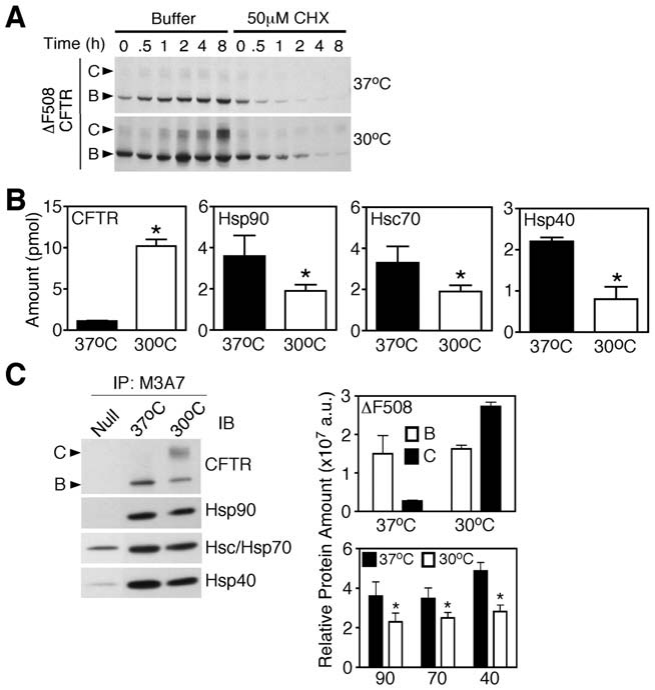
Quantification of ΔF508-CFTR interaction with core chaperones following temperature shift . **A.** Western blot analysis of HEK293 cells stably expressing ΔF508-CFTR cultured at 37°C or 30°C in the presence of 50 μM cyclohexamide (CHX) or vehicle control for the indicated time. **B.** Absolute quantification of ΔF508 CFTR and interacting chaperones at 37°C (black) or 30°C for 16 h (white). Absolute protein abundance of CFTR, Hsp90, Hsc70, and Hsp40 in CFTR-containing complexes is shown and expressed in pmols. **C.** Immunoblot and densitometric analysis for CFTR, Hsp90, Hsc/p70 and Hsp40 in CFTR-containing immunoprecipitates. In the densitometric analysis, the relative protein amount is shown in arbitrary units (a.u.). In all panels, data is shown as mean ± SD, n = 3 and asterisks represent p value <0.05 as determined by two-tailed t-test using the ΔF508-CFTR at 37°C sample as the reference.

To clarify when ΔF508-CFTR can be recovered in an export competent state, we next examined the effect of the protein synthesis inhibitor, cycloheximide (CHX), on cells incubated at 37°C and 30°C. At 37°C, we found that ΔF508 failed to exit the ER independent of the treatment condition with a substantial time-dependent loss of CFTR in the presence of CHX (**Fig.** **3A**). At 30°C, while ΔF508 was efficiently exported from the ER in the absence of CHX, no maturation of the ΔF508 pool synthesized at 37°C, was detected when cells were shifted to 30°C in the presence of CHX (**Fig.** **3A**). As a control, we did not observe any effect of CHX on the transport of the WT vesicular stomatitis virus glycoprotein (VSV-G) over the same time frame (data not shown). Because VSV-G and CFTR utilize the same exit signals to engage the COPII ER export machinery [34], [53], [54], it is unlikely that CHX interferes with the normal operation of the exocytic pathway. These data support the interpretation that synthesis of ΔF508 at physiological temperature (37°C) generates a folding intermediate that is irreversibly targeted for degradation (**Fig.** **2**) and only the pool of ΔF508-CFTR synthesized and folded at the permissive temperature is able to be exported.

To address the impact of reduced temperature on the folding of nascent ΔF508-CFTR, we examined the interaction of chaperones with ΔF508 following a 16 h shift to 30°C, a time period sufficient to re-establish the steady-state distribution of CFTR in the exocytic pathway [50] (**Fig.** **3C**). Consistent with the above data (**Fig.** **2**), ΔF508-CFTR was recovered in stoichiometric to supra-stoichiometric association with Hsps at 37°C, exhibiting a molar ratio of 1: 3.3±0.9: 3.0±0.8: 2.0±0.2 (total CFTR: Hsp90: Hsc70: Hsp40) (**Table** **2**). Following temperature-shift, CFTR levels increased 9-fold with >60% of the total ΔF508-CFTR pool being processed to band C (**Fig.** **3B & C**). The remaining pool was recovered in band B (**Fig.** **3C**). An analysis of ΔF508-CFTR complexes at both temperatures reveals a 2-fold decrease in the molar recovery of all chaperones at 30°C relative to 37°C (**Fig.** **3B** **&** **Table** **2**). When the absolute molar recovery of chaperones seen at 30°C was normalized to total CFTR pool, we obtained a molar ration of 1: 0.2±0.04: 0.2±0.04: 0.1±0.03 (total CFTR: Hsp90: Hsc70: Hsp40) (**Table** **2**), similar to the substoichiometric ratio we determined for the total pool of WT-CFTR (**Table** **1**). These observations are again supported by immunoblotting analyses, where the level of chaperones recovered with ΔF508-CFTR at 37°C, were reduced following low temperature rescue (**Fig. 3C**). An analysis of the stoichiometry of ΔF508-CFTR complexes still localized in the ER (band B) reveal a sub-stoichiometric association of chaperones with a ratio of 1: 0.4±0.1: 0.5±0.1: 0.2±0.1. Furthermore, an analysis of Hsp90 recovery with ΔF508-CFTR by SRM-MS after a 30 min and 6 h temperature rescue revealed a progressive shift from a stoichiometric recovery to a sub-stoichiometric recovery (data not shown). These data suggests that the low temperature rescue of ΔF508-CFTR alters the stoichiometric association of chaperones with the mutant in a manner similar to what is seen with WT-CFTR. These changes may, in part, reflect the slower folding and trafficking kinetics of ER export at 30°C due to energetic stabilization of the ΔF508 fold at reduced temperature (restoring WT-like interactions with PN components), and/or by changes to the PN composition or its function that may occur in response to incubation of cells at reduced temperature.

**Table 2:** Stoichiometry of the ΔF508 CFTR interaction with core chaperones at physiological and corrective temperatures.

	ΔF508 (37°C)	Molar Ratio to Total CFTR	ΔF508 (30^o^C)	Molar Ratio to Total CFTR	Fold Change
**CFTR**	1.1±0.1 pmol	1.0	10.2±1.7 pmol	1.0	+9.3±1.7
**Hsp90**	3.6±1.0 pmol	3.3±0.9	1.7±0.3 pmol	0.2±0.04	−2.1±0.7
**Hsp70**	3.3±0.8 pmol	3.0±0.8	1.9±0.3 pmol	0.2±0.04	−1.7±0.5
**Hsp40**	2.2±0.1 pmol	2.0±0.2	0.8±0.3 pmol	0.1±0.03	−2.8±1.0

Table depicting the absolute amounts of CFTR, Hsp90, Hsc70 and Hsp40, expressed in pmol. Also shown are the molar ratios of chaperones to total ΔF508-CFTR at both 37°C and 30°C. The fold change in the absolute amounts of CFTR, Hsp90 and Hsc70, expressed in pmol, relative to ΔF508-CFTR at 37°C is shown in the final column.

### Differential recognition of WT- and ΔF508-NBD1 by the Hsp90

**Figure 4:**
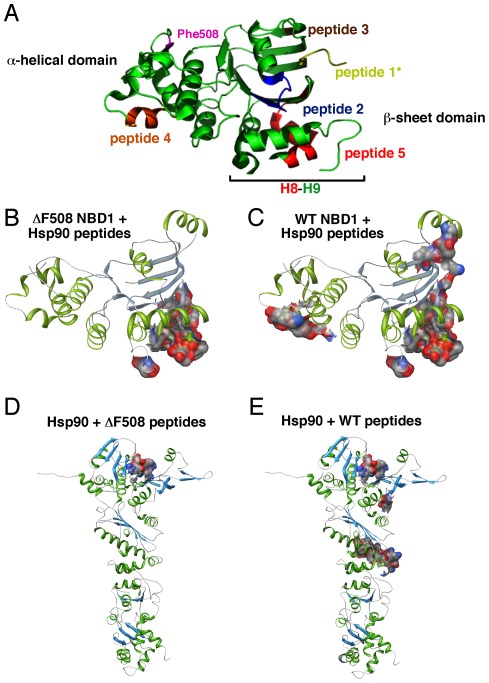
Structural mapping of the Interaction of NBD1 with Hsp90 using cross-linking. **A**. Ribbon diagram of NBD1 depicting Hsp90 interacting peptides. **B–C** Ribbon diagram of ΔF508-NBD1 (**B**) and WT-NBD1 (**C**) with associated Hsp90 interacting peptides shown as electrostatic map. **D–E.** Ribbon diagram of Hsp90 with associated ΔF508-NBD1 (**D**) and WT-NBD1 (**E**) interacting peptides shown as electrostatic map. Data shown is conserved peptides from 3 independent experiments.

In order to shed light on the differential ability of WT- and ΔF508-CFTR to resolve the Hsp-bound intermediate in the ER at physiological temperature, we examined their interactions *in vitro*. Purified Hsp90ß was incubated with either purified recombinant WT- or ΔF508-NBD1 in the presence of ATP. A zero-length cross-linker was used to capture the interactions and mass spectrometry was used to map the cross-linked peptides, using our recently described approach for defining the interaction of Hsp90 with its co-chaperone Aha1 [39]. Both ΔF508- and WT-NBD1 interacted with Hsp90 via a common binding site spanning the helix 8-helix 9 (H8–H9) region located at the C-terminus of the NBD1 domain (residues 624–643) (**Fig.** **4A** (peptide 5), **Fig. 4B, C& Fig. S3**). This common binding site represented the only interaction site between Hsp90 and ΔF508-NBD1, whereas WT-NBD1 exhibited four additional Hsp90 binding sites (**Fig** **4A, C** **& Fig S3**). Three of these additional sites, as well as the H8–H9 binding site, locate to the NBD1-NBD2 dimerization interface, which includes the Walker A motif region (residues 453–464), which is critical for ATP binding and formation of a stable NBD1-NBD2 dimer (**Fig.** **4A** (peptide 2), **Fig.** **4C** **& Fig. S3**). The last unique site of Hsp90 binding to WT-NBD1 is located on the opposite face of the domain, in close proximity to the F508 residue (residues 526–532) (**Fig.** **4A** (peptide 4), **Fig.** **4C** **& Fig. S3**). Gel filtration chromatography did not reveal aggregation of the ΔF508-NBD1 domain (data not shown), suggesting that the reduced number of interaction sites with Hsp90 seen with the mutant NBD1 are not due to a lower availability of this protein. This *in vitro* data highlights the impact of the ΔF508 mutation on the folding of NBD1, suggesting that the structural differences between the WT and ΔF508-NBD1 impede the ability of the mutant variant to properly engage the Hsp90 chaperone system. Interestingly, whereas the H8–H9 helices of both WT- and ΔF508-NBD1 interacted with the N-terminal domain of Hsp90 (**Fig.** **4D** **& Fig. S3**), WT-NBD1 also interacted with the middle domains of Hsp90 via the additional NBD1 interaction sites (**Fig.** **4E** **& Fig. S3**). Since both regions of Hsp90 have previously been implicated in client interactions [4], these data show that Hsp90 may require the additional contact sites found in WT-NBD1 to properly engage this client in order to complete the folding cycle, a step that is impaired in the absence of F508. We suggest that the inability of ΔF508 to properly integrate with the Hsp90 chaperone machinery to complete its folding cycle and trigger chaperone release is a major contributing factor to the inefficient ER export of ΔF508 by COPII and contributes to its efficient targeting for ERAD.

## Discussion

Although Hsp90 has previously been shown to qualitatively participate in the biogenesis of CFTR [33], [38], [39], a quantitative description of how the WT and mutant CFTR engage the PN in health and disease has been elusive. To address this concern, we have focused our attention on the role of the PN in managing the ΔF508 mutant fold. Using quantitative SRM-MS, we have now characterized a novel folding step(s) that occurs as a result of the altered ability of ΔF508-CFTR to interact with PN components critical for its biogenesis. We observed that the ΔF508 mutant accumulates *in vivo* in a stalled folding intermediate(s) containing stoichiometric to supra-stoichiometric core components of the Hsp folding system. Previous analyses of purified NBD1 and full length CFTR have revealed structural defects associated with the F508 deletion [25], [31], [48], [55]. We have now demonstrated the consequence of these structural differences *in vitro*- an altered recognition by Hsp90 of WT and mutant NBD1. Together, our *in vivo* and *in vitro* analyses provide new insights into the key role of chaperones in CFTR WT and disease biology, and highlights the importance of quantitatively characterizing folding intermediates using SRM-MS technologies in order to understand the role of the PN in human misfolding disease.

Our finding reveal that the deletion of F508 alters the ability of the NBD1 to interact with Hsp90 *in vitro* provides new insight into possible steps that contribute to disease. We found that the H8–H9 region of NBD1 is the only common site of Hsp90 recognition between WT NBD1 and ΔF508 NBD1. In contrast, Hsp90 interacted with WT-NBD1 through 4 additional binding sites that localize to the NBD2 dimerization interface and the α-helical domain of NBD1. Specifically, the middle domain of Hsp90 binds to peptide 4, which localizes to the recently identified folded core of NBD1 [20], a region thought to be critical for the stability and subsequent folding of WT-CFTR [20], [21], [23], [25], [26], [28]–[30], [56]. This stable core structure is never achieved in ΔF508-NBD1, unless the I539T and/or other suppressor mutations are introduced [20], [21], [23], [29], [30]. Interestingly, peptide 4 spans residues 525–532 of NBD1, which places it in close proximity to the I539T mutation and within the stable core of the WT protein. While the binding interactions observed with Hsp90 *in vitro* used purified, recombinant NBD1, previous evidence strongly suggests the importance of Hsp90 for CFTR function *in vivo* [33], [38], [39]. We now raise the possibility that the inability of ΔF508-NBD1 to achieve its native fold *in vivo* is reflected in the altered Hsp90 recognition observed *in vitro*. This altered recognition could favor targeting of ΔF508 CFTR for degradation and/or prevent trafficking of ΔF508-CFTR from the ER.

In addition to characterizing the differential binding of Hsp90 to WT- and ΔF508-NBD1 *in vitro*, our quantitative SRM-MS analysis has revealed the stoichiometries of Hsp chaperone complexes binding full-length CFTR *in vivo*. The ER localized ΔF508-CFTR complexes (band B) at 37°C exhibited chaperone stoichiometries of 1∶2∶2 to 1∶3∶3 (CFTR: Hsp90: Hsc70), suggesting that at least a functional dimer of Hsp90 and several (2–3) Hsp70 monomers are bound to each molecule of ΔF508 CFTR. In contrast, we recovered the total pool of WT CFTR (bands B plus C) at a chaperone stoichiometry of 1∶0.2∶0.2 (CFTR: Hsp90: Hsc70) at 37°C, leading us to suggest that WT CFTR matures through early chaperone-dependent steps defined by the stoichiometries potentially associated with band B WT CFTR. Thus, we suggest that ΔF508 accumulates in a stalled folding intermediate, the chaperone trap, impeding its progression along the folding and trafficking axis, resulting in its targeting for degradation. Consistent with this interpretation, our results also directly demonstrate that reduced temperature rescue of ΔF508-CFTR *in vivo* can resolve this stalled Hsp-bound intermediate, leading to restoration of the sub-stoichiometric Hsp chaperone association observed for the WT protein *in vivo*. One possibility is that reduced temperature provides thermodynamic stability to the nascent ΔF508-NBD1 fold, partially restoring folding and, hence, WT-like interactions with PN components critical to its biogenesis. Alternatively, reduced temperature may change the composition of the PN and/or the properties of existing PN components, altering their interactions with ΔF508-CFTR and thereby promoting folding and trafficking. These potential interpretations are consistent with the effect of siRNA silencing of Aha1 [33], where we found that altering the ATPase activity of Hsp90 can restore function at the cell surface. Altering the PN to restore function is consistent with the observed effects of PN modulating proteostasis regulators, [2], [11], [12], on the activity of the UPR [57] and the HSR [58], [59] signaling pathways, that can be used to correct a wide range of protein folding problems [8], [60]–[76]. Although our studies highlighting the interaction of NBD1 with Hsp90 were performed *in vitro* with purified proteins, we suggest that the structural interactions observed in these *in vitro* experiments are reasonable candidates for the structural interactions occurring *in vivo* with full-length CFTR contributing to the chaperone trap state.

**Figure 5:**
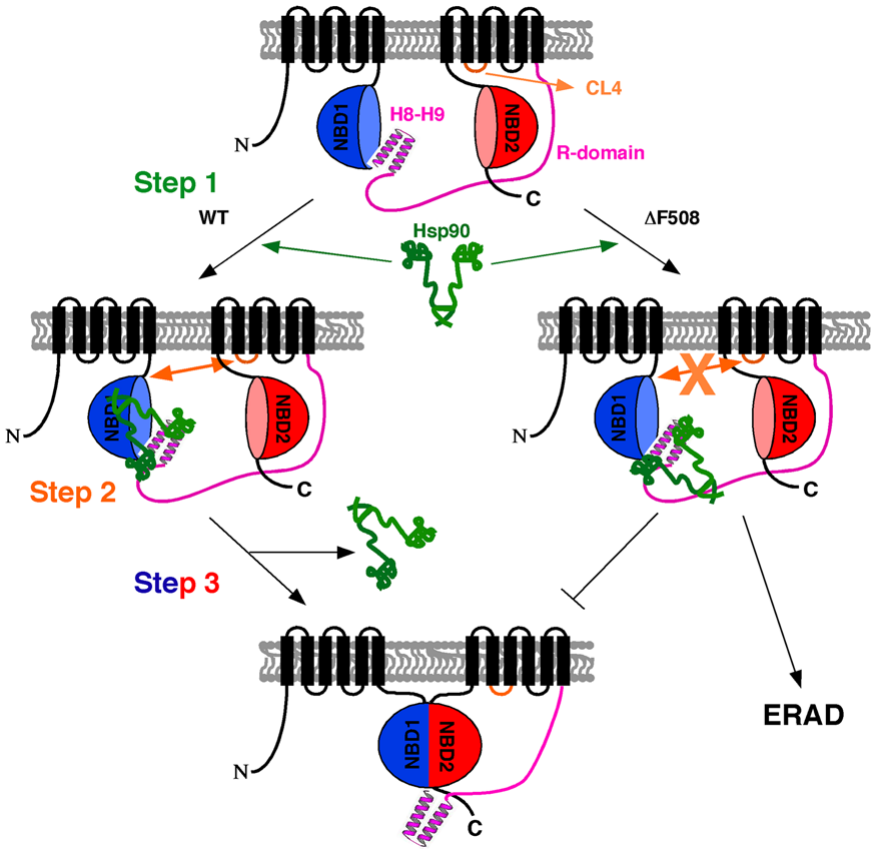
Minimal sequential ordering of intra- and inter-domain folding events responsible for CFTR folding and trafficking. Intra-domain folding of NBD1 is dictated by the Hsp90 system (step 1). A structural rearrangement occurs in response to the binding of cytoplasmic loop 4 (CL4) to the F508 containing hydrophobic pocket present WT NBD1 (step 2). The binding of CL4 provides a stabilizing effect on NBD1, releasing Hsp90 and promoting H8–H9 helix-coil transition. This H8–H9 transition would expose the NBD2-binding interface of NBD1 and allow NBD1 to ‘chaperone’ *in trans* the folding of NBD2 (step 3).

Our studies lead us to propose a minimal sequential ordering of intra- and inter-domain folding events that manage the biogenesis of WT-CFTR, which fails in human ΔF508 disease (**Fig.** **5**). Here, intra-domain folding of WT NBD1 could involve a stoichiometric interaction with the Hsc/p70-Hsp90 chaperone system, as shown herein (**Fig.** **5**, step 1). This is consistent with the many descriptions of the role of the Hsc/p70-Hsp90 chaperone system in the nascent protein folding of other proteins [9], [32], [33], [38], [39], [64], [66], [68], [77]–[87]. A recent analysis of the crystal structure of NBD1 reveals that the H8–H9 helices, recognized by Hsp90 (**Fig.** **4**), would have to be re-oriented in order to promote dimerization and post-translational chaperoning of NBD2 [88]–[91]. NMR studies have also shown that such a structural rearrangement occurs in response to the binding of cytoplasmic loop 4 (CL4), a short loop that links helices 10 and 11 in TMD2 (shown in orange in **Fig** **5**), to the F508 containing hydrophobic pocket present in WT NBD1 [88]–[91]. Because NBD1 interacts with CL4, one possibility is that Hsp90, when properly engaged, generates a stabilized folding intermediate capable of CL4 binding (**Fig.** **5**, step 2). Together with the reported effects of the ΔF508 mutation on interactions with additional TMD loops [89]–[92], and the folding and stability of NBD2 [48], the binding of CL4 could, as suggested by others [89]–[94], contribute to the subsequent stabilization of NBD1, potentially resolving the Hsp90 interaction and promoting H8–H9 helix-coil transition. The release of Hsp90 and the reorientation of the H8–H9 helices would now expose the NBD2-binding interface of NBD1 allowing for NBD1-mediated chaperoning of NBD2 (**Fig.** **5**, step 3) [21], [29]. Although both WT- and ΔF508-NBD1 interact with Hsp90 via the H8–H9 region, we suggest that ΔF508 may be unable to achieve the CL4 binding competent intermediate, possibly due to the inability of the mutant to properly engage the Hsp chaperone system at the additional binding sites shown *in vitro* that includes the stable folded core that occurs during folding of WT-NBD1 (**Fig.** **4**). This results in trapping of the ΔF508-CFTR in the stalled Hsp90-dependent folding step observed herein. Whether this is an on-pathway or off-pathway intermediate remains to be shown, particularly given the complexity of the Hsp90 chaperone system, which could have different kinetic and/or thermodynamic branch points sensitive to the progress of the folding reaction. Failure to resolve this Hsp-bound stalled intermediate would likely interfere with subsequent inter-molecular interactions, such as NBD2 dimerization and subsequent folding of full length CFTR (**Fig.** **5**).

While further experiments are required, the stalled folding intermediate may direct ΔF508 along an alternative pathway responsible for degradation by the proteasome [3], [17]–[19] (**Fig.** **5**). We now suggest that targeting the PN through proteostasis regulation [2], [11], [12], [33], [95] may alter the interaction of ΔF508-CFTR with the stalled Hsp90 chaperone system [1], [12]. One possibility is that by managing the activity of the ATPase regulators of Hsp90 and Hsc/p70 by Aha1 (Hsp90) [33] or Hsp40 isoforms [37], respectively, we will create a folding environment that stabilizes ΔF508-NBD1 of CFTR for subsequent folding steps involving downstream domains such as NBD2, thereby enhancing exit from the ER. Alternatively, given that ΔF508-CFTR is very often detected in an unstable band C form at the cell surface, proteostasis regulation could contribute to a new chaperone environment that stabilizes this cell surface localized species, enhancing channel function. Thus, by managing the composition and/or activity of the PN, we may promote both assembly and stability of CFTR. Proteostasis regulation could have an important impact on mitigating CF in the clinic.

## Materials and Methods

### Preparation and purification of ^15^N-labeled proteins

Human Hsp90β, Hsc70 (heat shock cognate 71 kDa, isoforms 1 and 2), and Hsp40 heat shock 40 kDa (protein 4), were cloned into pET11d vector containing a hexa-histidine tag. The plasmids were transformed into *E. coli* (strain BL21) and grown to an OD between 0.8–1.0 in >98% ^15^N-atom Spectra 9 labeling media (Spectra Stable Isotopes). Protein expression was induced with 1 mM IPTG at 18°C overnight. Cells were harvested and lysed by sonication. The cleared supernatants were subsequently applied to a 1 ml HisTrapHP chelating column (GE Healthcare), charged with nickel sulfate controlled by an AKTA FPLC system (GE Healthcare) using the following buffer system: IMAC Buffer A (50 mM KH_2_PO_4_ pH 8.0, 500 mM KCl, 5 mM ß-mercaptoethanol; IMAC Buffer B (50 mM KH_2_PO_4_ pH 8.0, 500 mM KCl, 1 M Imidazole, 5 mM ß-mercaptoethanol). Bound proteins were eluted with imidazole and further purified by anion exchange chromatography on a 1 ml MonoQ HR 5/5 column (GE Healthcare) using the following buffer system: AE Buffer A (20 mM Tris pH 7.5, 1 mM Mg-acetate, 1 mM DTT); AE Buffer B (20 mM Tris pH 7.5, 1 mM MgAcetate, 1 M NaCl, 1 mM DTT). Hsc70, and Hsp90 were further purified using gel filtration using a 20 ml Superose 6 HR 10/30 column (GE Healthcare) in 50 mM Hepes pH 7.2, 500 mM NaCl, 1 mM MgCl2, 1 mM DTT. The purified proteins were then dialyzed against 25 mM Hepes pH 7.2, 125 mM K-acetate. The resulting proteins were tested for purity using silver stained gels and mass spectrometry. The percent atomic enrichment was estimated as previously described [96]–[98].

### Immunoblotting

A dilution series of ^14^N recombinant, purified Hsc70 (isoform 1 and 2), Hsp90β and HEK293 lysates were analyzed by Western immunoblotting with rabbit polyclonal heat shock protein 90 antibody (Stressgen SPA-846) (1.20 000) and rabbit polyclonal antibody recognizing both Hsp70 and Hsc70 (Stressgen SPA-812) (1∶50,000). Chemiluminescent detection was performed with ECL, using goat anti-rabbit IgG horse radish peroxidase (1∶10,000) (Pierce). Densitometric analysis was performed using Image J software.

### Sample preparation for validation experiments

Three labeled AQUA peptides for CFTR (ADLINNGTIAK (^13^C^15^N), SFYPEEVSSMVLTK (^13^C^15^N), and GQLLAVSTGAGK(^13^C^15^N)) were purchased from Sigma Genosys, and dissolved in formic acid to a final concentration of 5 pmol/µl. An aliquot of HEK293 cell lysate (50 μg) was mixed with 2 µg of ^15^N labeled Hsp40, Hsc70 and Hsp90 proteins, a second 50 µg aliquot of HEK293 cell lysate was mixed with 5 pmols/µl of the labeled AQUA peptides SFYPEEVSSMVLTK (Hsc70) and ADLINNGTIAK (Hsp90), and a 2 µg aliquot of HEK293 cell lysate was retained for Western blot analysis. These analyses were performed in triplicate. For TSQ analysis 5 µg of lysate was mixed with 0.6 µg and 0.2 µg of ^15^N labeled Hsp40, Hsc70 and Hsp90, respectively. Analysis was also performed in triplicate.

### Cell culture

HEK293 cell lines either not expressing or stably expressing either WT or ΔF508-CFTR was kindly provided by Dr. Neil A. Bradbury (University of Pittsburgh School of Medicine, Pittsburgh, PA 15261) and grown in DMEM supplemented with 10% FBS and Pen/Strep plus 150 μg/ml hygromycin B at 37°C. Temperature shift experiments were performed by placing plates at 30°C for 16 h.

### Anti-CFTR coupling to beads

Monoclonal antibody (M3A7) against an epitope at the C-terminal end of NBD2 [99] was coupled to Gamma Bind Beads (Amersham) at final concentration of 4 mg/ml using dimethylpimelimidate (Pierce 21667). In brief, 2–4 mg per ml of wet beads were incubated with the appropriate amount of M3A7 antibody at room temperature for 1 h with rocking. The beads were washed once with 10 volumes of 0.1 M sodium borate pH 9 by a brief centrifugation (3000×g for 5 min) and subsequently resuspended in 10 volumes of 0.1 M sodium borate pH 9. The crosslinker, dimethylpimelimidate (DMP) (Pierce 21667) was added in solid form to a final concentration of 20 mM and mixed by rocking for 30 min at room temperature. The beads were subsequently washed once with 0.2 M ethanolamine (pH 8) and remaining free crosslinker was quenched by incubating the beads for 2 h at room temperature in 0.2 M ethanolamine. The beads were washed with three changes of PBS pH 7.4 and resuspended in PBS containing 0.02% sodium azide as a preservative and stored at 4°C.

### CFTR Immunoprecipitation

HEK293 cells expressing either WT- or ΔF508-CFTR were washed with 2 changes of ice cold PBS, and lysed in the dish on ice using lysis buffer (25 mM TRIS pH 7.4, 150 mM NaCl, 1% Triton X 100, protease inhibitor at 2 mg/ml final concentration (Roche 11836145001)) at 15 μl lysis buffer/cm^2^. Cell lysates (1 mg total protein) were incubated with 60 µl of 50% slurry of M3A7 Sepharose beads overnight at 4°C. The beads were subsequently washed with 3 changes of 300 μl of wash buffer (50 mM Tris pH 7.4/150 mM NaCl) and bound protein eluted in elution buffer (50 mM Tris pH 6.8, 1% SDS). Eluted proteins were either methanol/chloroform precipitate and subjected to tryptic digestion and mass spectrometric analysis or subject to Western blot analysis as described above. The analyses comparing WT versus ΔF508 and ΔF508 at 37°C vs ΔF508 at 30°C are paired and done separately.

### Purification of NBD1

Recombinant human WT- and ΔF508-NBD1 were a kind gift from Dr Gergely Lukacs (McGill University, Department of Physiology, Montreal, Quebec) and were purified as previously described [100]. The domain includes residues 389–678 for WT-NBD1 and 389–677 for ΔF508-NBD1 and contains both the regulatory insertion (RI) (404–435) and the regulatory extension (RE) (639–670). The sequences are listed below.

*NBD1-WT(F494N)-*TTEVVMENVTAFWEEGFGELFEKAKQNNNNRKTSNGDDSLFFSNFSLLGTPVLKDINFKIERGQLLAVAGSTGAGKTSLLMMIMGELEPSEGKIKHSGRISFCSQNSWIMPGTIKENIIFGVSYDEYRYRSVIKACQLEEDISKFAEKDNIVLGEGGITLSGGQRARISLARAVYKDADLYLLDSPFGYLDVLTEKEIFESCVCKLMANKTRILVTSKMEHLKKADKILILHEGSSYFYGTFSELQNLQPDFSSKLMGCDSFDQFSAERRNSILTETLHRFSLEGDAPVS.

*NBD1-ΔF508 (F494N)*-TTEVVMENVTAFWEEGFGELFEKAKQNNNNRKTSNGDDSLFFSNFSLLGTPVLKDINFKIERGQLLAVAGSTGAGKTSLLMMIMGELEPSEGKIKHSGRISFCSQNSWIMPGTIKENIIGVSYDEYRYRSVIKACQLEEDISKFAEKDNIVLGEGGITLSGGQRARISLARAVYKDADLYLLDSPFGYLDVLTEKEIFESCVCKLMANKTRILVTSKMEHLKKADKILILHEGSSYFYGTFSELQNLQPDFSSKLMGCDSFDQFSAERRNSILTETLHRFSLEGDAPVS.

### Crosslinking of Hsp90 with NBD1

A mixture of 10 μM purified, recombinant human Hsp90β was mixed with 10 μM of purified, recombinant human WT-NBD1 or ΔF508-NBD1 in 25 mM HEPES, pH 7.4, 100 mM NaCl and allowed to bind on ice for 30 min. A freshly prepared mixture of EDC and Sulfa-NHS were added to the protein solution and incubated for 30 min at room temperature to crosslink the bound proteins. Excess crosslinker was subsequently quenched by the addition of Tris-HCl and incubation at room temperature for an additional 15 min. The proteins were then acetone-precipitated and trypsin-digested for mass spectrometric (MS) analysis as described previously [27].

### Digestion

The precipitated eluates from above were resuspended in 100 mM Tris pH 8.5 and mixed with 0.3 µg Hsp90, 1 µg Hsp70, 1 μg Hsp40 and 5 pmols CFTR AQUA peptide. The samples were denatured by addition of solid urea to a final concentration of 8 M. The sample was reduced with 3 mM TCEP (Sigma) for 20 min at room temperature and subsequently alkylated with 10 mM iodoacetamide (Sigma) for 30 min at room temperature. The samples were then digested overnight with trypsin by adding 1/50 total volume of trypsin solutiion (Protégé).

### Mass spectrometry

Using multidimensional protein Identification technology (MudPIT) [55]–[57] the protein digest was pressure-loaded onto a fused silica capillary desalting column containing 5 cm of 5 µm Polaris C18-A material (Metachem, Ventura, CA) packed into a 250-µm i.d capillary with a 2 µm filtered union (UpChurch Scientific, Oak Harbor, WA). The desalting column was washed with buffer containing 95% water, 5% acetonitrile, and 0.1% formic acid. After desalting, a 100-µm i.d capillary with a 5-µm pulled tip packed with 10 cm 3-µm Aqua C18 material (Phenomenex, Ventura, CA) followed by 3 cm 5-µm Partisphere strong cation exchanger (Whatman, Clifton, NJ) was attached to the filter union and the entire split-column (desalting column–filter union–analytical column) was placed inline with an Agilent 1100 quaternary HPLC (Palo Alto, CA) and analyzed using a modified 12-step separation described previously [26]. The buffer solutions used were 5% acetonitrile/0.1% formic acid (buffer A), 80% acetonitrile/0.1% formic acid (buffer B), and 500 mM ammonium acetate/5% acetonitrile/0.1% formic acid (buffer C). Step 1 consisted of a 100 min gradient from 0–100% buffer B. Steps 2–5 had the following profile: 3 min of 100% buffer A, 2 min of X% buffer C, a 10 min gradient from 0–15% buffer B, and a 97 min gradient from 15–45% buffer B. The 2 min buffer C percentages (X) were 15, 30, 40, 60% respectively for the 5-step analysis. The final step, the gradient contained: 3 min of 100% buffer A, 20 min of 100% buffer C, a 10 min gradient from 0–15% buffer B, and a 107 min gradient from 15–70% buffer B. As peptides eluted from the microcapillary column, they were electrosprayed directly into an LTQ dimensional ion trap mass spectrometer (ThermoFinnigan, Palo Alto, CA) with the application of a distal 2.4 kV spray voltage. Following a cycle of one full-scan mass spectrum (400–1400 m/z), parent ions were selected for collision at 1.5 m/z width in data independent mode at 35% normalized collision energy. This was repeated continuously throughout each step of the multidimensional separation. The validation experiment reported in the text of the Results was performed on both LTQ and a TSQ Quantum Ultra, a triple quadripole instruments to test the validity of the approach with a resulting high correlation of absolute measurements. Substantially less starting material and a shorter separation gradient was used in TSQ analysis, highlighting its increased sensitivity for this technology.

### TSQ reverse phase analysis

In the TSQ Quantum ultra (ThermoElectron, San Jose, CA), peptides were monitored by single-ion reaction monitoring (SRM) mass spectrometry (SRM-MS) as described in the Results. Precursor and fragment ion selection and collision energies were empirically determined from LTQ data fragmentation data and TSQ analysis of purified proteins. The digested peptides were loaded under pressure onto a reverse phase column (100 µm capillary loaded with 10 cm of 5 u AQUA C18) and a Surveyor™ MS Pump was used to produce and deliver a solvent gradient (A: 0.1%FA/5%ACN) (B: 0.1%FA/80%%ACN) The flow rate was 200 nL/min with the use of a flow splitter. The linear ramp was from 15% B to 100% B in 50 min. Quantum Q1 was kept at 0.7 m/z and Q3 at 0.7 m/z with a scan time of 0.1 seconds and 0.02 scan width.

### Data analysis

Tandem mass spectra were searched as described [55]–[57] using the SEQUEST algorithm against an EBI-IPI human 3.05 04-04-2007 database concatenated to a decoy database in which the sequence for each entry in the original database was reversed. The validity of peptide/spectrum matches was assessed using the SEQUEST-defined parameters, cross-correlation score (XCorr), and normalized difference in cross-correlation scores (DeltaCn). SEQUEST results were assembled and filtered using the DTASelect (version 2.0) program. DTASelect 2.0 uses a quadratic discriminant analysis to dynamically set XCorr and DeltaCN thresholds for the entire data set to achieve a user specified false positive rate (5% in this analysis). The false positive rates are estimated by the program by ratioing the number and quality of spectra to the decoy database. For ^15^N enrichment calculations a published algorithm was used, based on information from high-resolution scans which determined the ^15^N peptide isotopic distributions, which are characteristic of the ^15^N atomic percent enrichment of the peptide.

### Calculation of peptide and protein ratios extraction of chromatograms

After filtering the results from SEQUEST using DTASelect2 ion chromatograms were generated from tandem mass spectra using a program CENSUS [101]. This was used to calculate peptide ion intensity ratios for each pair of extracted ion chromatograms. Several spectra were manually validated. For TSQ data peaks area ratios were determined manually in Xcalibur or using Thermo Finnigan software LC Quant.

## Supporting Information

Figure S1 **Validation of absolute quantification method for rescue of CFTR**. (**A**) Absolute abundances of Hsp90 peptides (ng/µl) calculated by SRM-MS: (1) ELISNASDALDK (2) ELISNANSDALDKIR ((3) ADLINNLGTIAK (4) NPDDITNEEYGEFYK. (**B**) Absolute abundances of Hsc70 peptides (ng/µl) calculated by SRM-MS: (1) NQVAMNPTNTVFDAK (2) SFYPEEVSSMVLTK (3) SQIHDIVLVGGSTR (4) SINPDEAVAYGAAVQAAILSGDK. All panels, data is shown as mean ± SD, n = 3.
(TIF)

Figure S2 **▵F508 fails to exist the ER when bound to chaperones at physiological temperatures.** Western blot analysis of CFBE cells treated with 50 μM MG132 or vehicle control for 6 h at 37°C. Data shown is the mean ± SD, n = 3.
(TIF)

Figure S3 **Peptides recovered in cross-linking experiments.** Consensus peptides recovered in the cross-linking of Hsp90 with WT- or ΔF508-NBD1. The color of the respective peptides is in accordance with the highlighted peptides in **Fig.** **4A**. The location of these peptides in WT- and ΔF508-CFTR are illustrated in the sequence. The corresponding Hsp90 cross-linked peptide is also indicated in matching color in the included sequence.
(TIF)
