# Supplementary figures and images for: A Chaperone Trap Contributes to the Onset of Cystic Fibrosis
Source: PLoS One. 2012 May 31;7(5):e37682. doi: 10.1371/journal.pone.0037682 (PMC3365120; doi:10.1371/journal.pone.0037682)

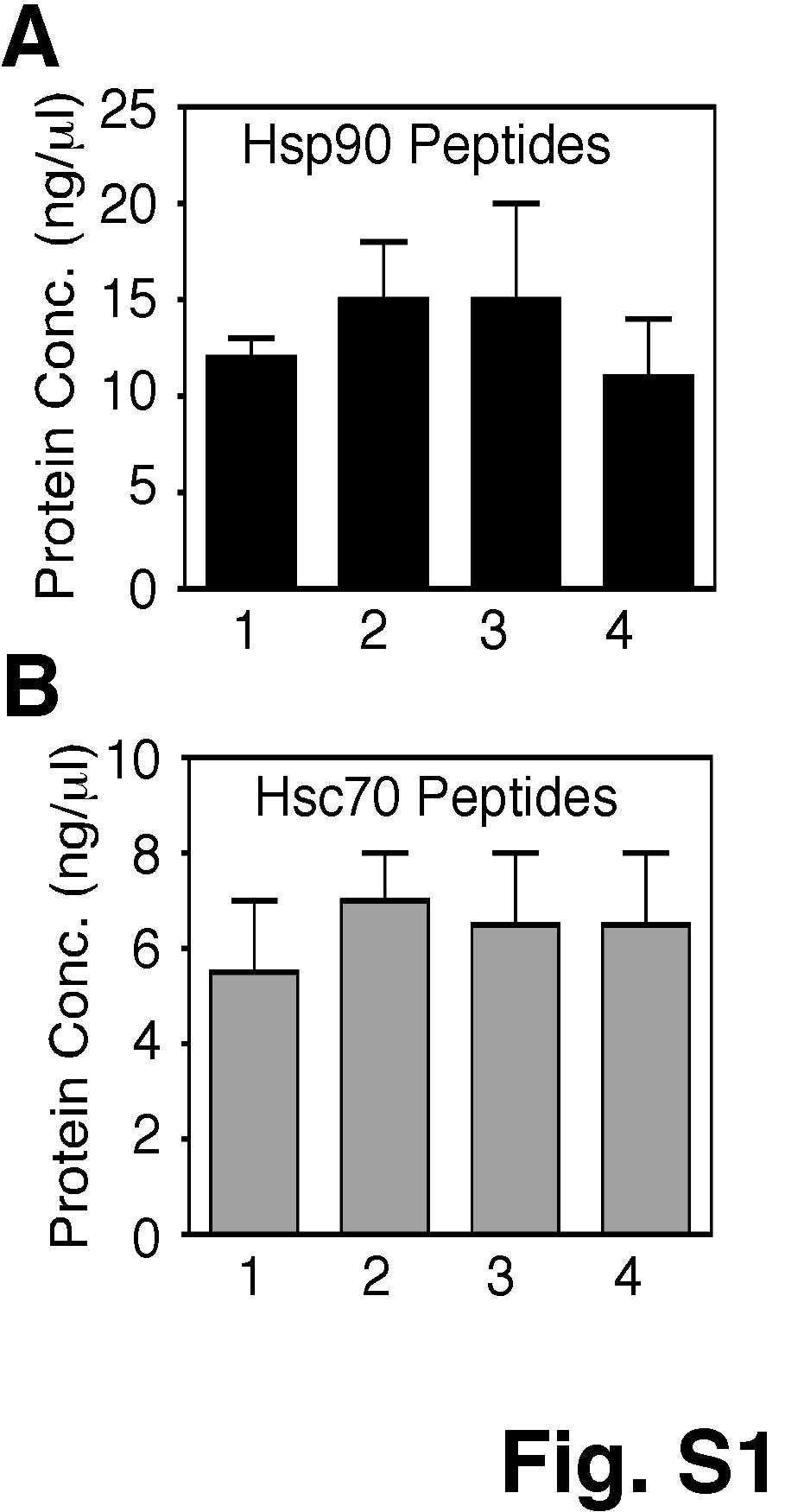

Supplement: Figure S1 — Validation of absolute quantification method for rescue of CFTR. (A) Absolute abundances of Hsp90 peptides (ng/µl) calculated by SRM-MS: (1) ELISNASDALDK (2) ELISNANSDALDKIR ((3) ADLINNLGTIAK (4) NPDDITNEEYGEFYK. (B) Absolute abundances of Hsc70 peptides (ng/µl) calculated by SRM-MS: (1) NQVAMNPTNTVFDAK (2) SFYPEEVSSMVLTK (3) SQIHDIVLVGGSTR (4) SINPDEAVAYGAAVQAAILSGDK. All panels, data is shown as mean ± SD, n = 3. (TIF) [file pone.0037682.s001.tif]

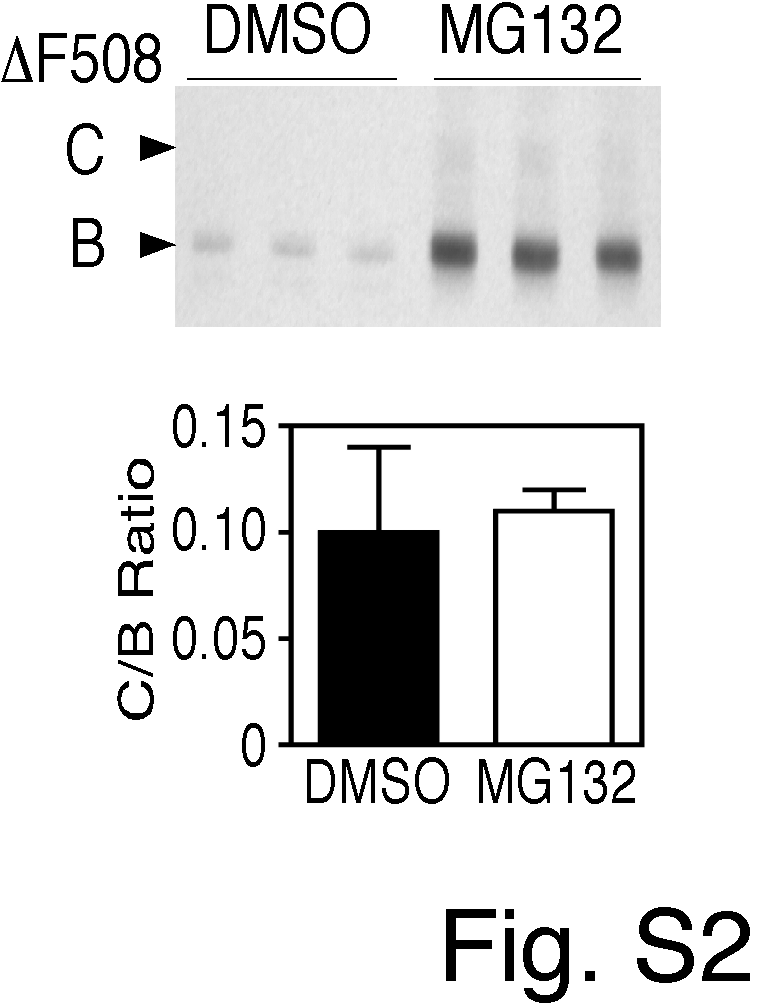

Supplement: Figure S2 — ▵F508 fails to exist the ER when bound to chaperones at physiological temperatures. Western blot analysis of CFBE cells treated with 50 μM MG132 or vehicle control for 6 h at 37°C. Data shown is the mean ± SD, n = 3. (TIF) [file pone.0037682.s002.tif]

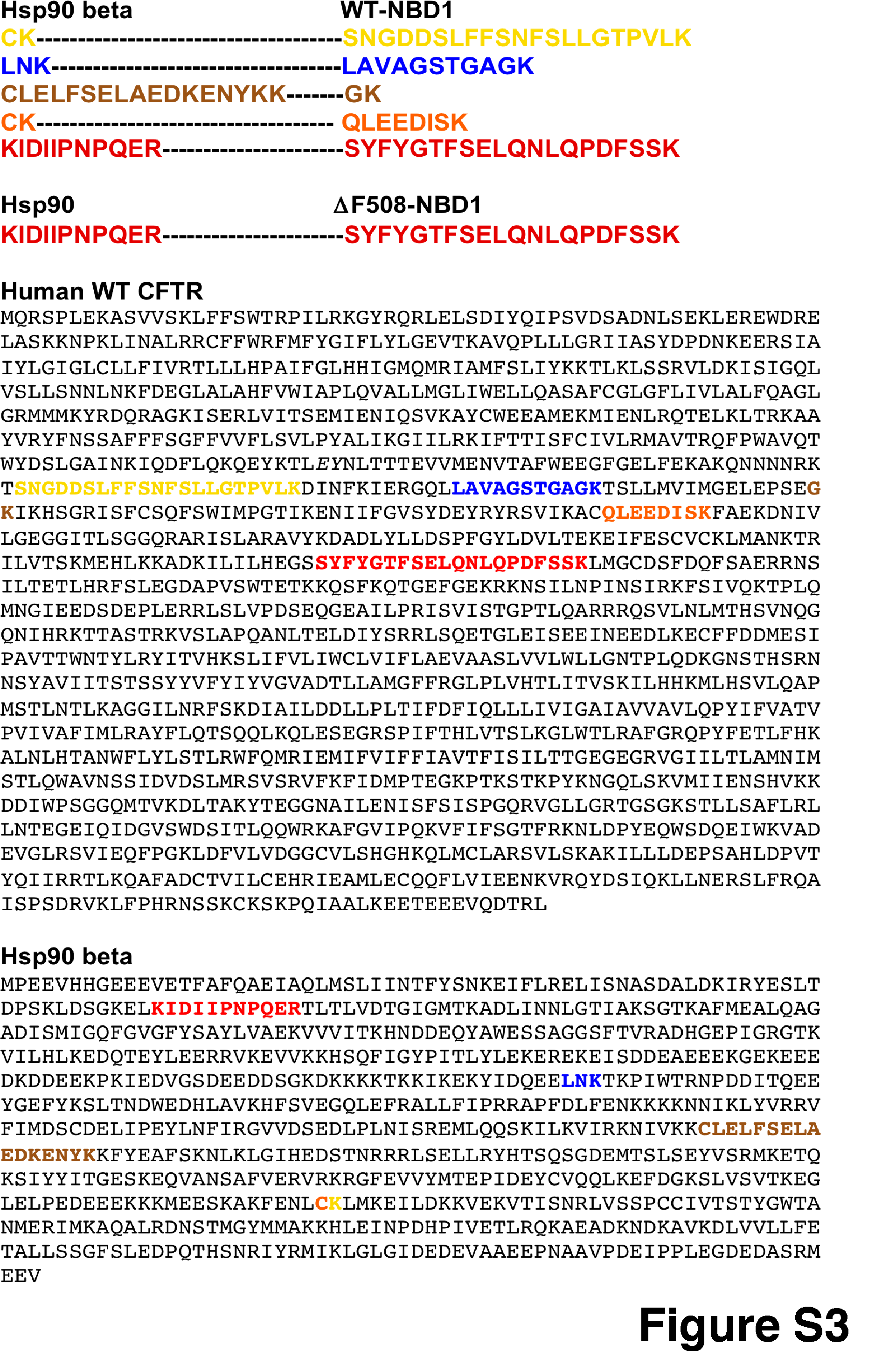

Supplement: Figure S3 — Peptides recovered in cross-linking experiments. Consensus peptides recovered in the cross-linking of Hsp90 with WT- or ΔF508-NBD1. The color of the respective peptides is in accordance with the highlighted peptides in Fig. 4A . The location of these peptides in WT- and ΔF508-CFTR are illustrated in the sequence. The corresponding Hsp90 cross-linked peptide is also indicated in matching color in the included sequence. (TIF) [file pone.0037682.s003.tif]
